# Detrimental Effects of β2‐Microglobulin on Muscle Metabolism: Evidence From In Vitro, Animal and Human Research

**DOI:** 10.1002/jcsm.13745

**Published:** 2025-03-03

**Authors:** Shibo Wei, So Jeong Park, Eunah Choi, Il‐Young Jang, Yan Zhang, Yingqi Xue, Yunju Jo, Hee‐Won Jung, Eunhye Ji, Jin Young Lee, Yujin Moon, Eunju Lee, Dongryeol Ryu, Beom‐Jun Kim

**Affiliations:** ^1^ Department of Biomedical Science and Engineering Gwangju Institute of Science and Technology Gwangju Republic of Korea; ^2^ Asan Institute for Life Sciences, Asan Medical Center University of Ulsan College of Medicine Seoul Republic of Korea; ^3^ Division of Geriatrics, Department of Internal Medicine, Asan Medical Center University of Ulsan College of Medicine Seoul Republic of Korea; ^4^ Division of Endocrinology and Metabolism, Department of Internal Medicine, Asan Medical Center University of Ulsan College of Medicine Seoul Republic of Korea; ^5^ Sarcopenia Total Solution Center Wonkwang University School of Medicine Iksan Republic of Korea

**Keywords:** ageing, biomarker, pro‐ageing factor, sarcopenia, β2‐microglobulin

## Abstract

**Background:**

β2‐Microglobulin (B2M) has garnered considerable interest as a potential pro‐ageing factor, leading to speculation about its involvement in muscle metabolism and the development of sarcopenia, a key component of ageing phenotypes. To explore this hypothesis, we conducted a comprehensive investigation into the impact of B2M on cellular and animal muscle biology, as well as its clinical implications concerning sarcopenia parameters in older individuals.

**Methods:**

In vitro myogenesis was induced in mouse C2C12 myoblasts with 2% horse serum. For in vivo research, C57BL/6 mice aged 3 months were intraperitoneally given 250 μg of B2M daily, and muscular alterations were assessed one month later. Human blood samples were obtained from 158 participants who underwent assessments of muscle mass and function at an outpatient geriatric clinic affiliated with a teaching hospital. Sarcopenia and associated parameters were assessed using cut‐off values specifically tailored for the Asian population. The concentration of serum B2M was quantified through an enzyme‐linked immunosorbent assay.

**Results:**

Recombinant B2M inhibited in vitro myogenesis by increasing intracellular reactive oxygen species (ROS) production. Furthermore, B2M significantly induced differential myotube atrophy via ROS‐mediated ITGB1 downregulation, leading to impaired activation of the FAK/AKT/ERK signalling cascade and enhanced nuclear translocation of FoxO transcription factors. Animal experiments showed that mice with systemic B2M treatment exhibited significantly smaller cross‐sectional area of tibialis anterior and soleus muscle, weaker grip strength, shorter grid hanging time, and decreased latency time to fall off the rotating rod, compared to untreated controls. In a clinical study, serum B2M levels were inversely associated with grip strength, usual gait speed and short physical performance battery (SPPB) total score after adjustment for age, sex, and body mass index, whereas sarcopenia phenotype score showed a positive association. Consistently, higher serum B2M levels were associated with higher risk for weak grip strength, slow gait speed, low SPPB total score, and poor physical performance.

**Conclusion:**

These results provide experimental evidence that B2M exerted detrimental effects on muscle metabolism mainly by increasing oxidative stress. Furthermore, we made an effort to translate the results of in vitro and animal research into clinical implication and found that circulating B2M could be one of blood‐based biomarkers to assess poor muscle health in older adults.

## 1 Introduction

The growing number of individuals in the senior population and advancements in healthcare have sparked interest in finding efficient ways to reverse or slow ageing process. Although a number of factors, including oxidative stress, cellular senescence and dysregulation of energy homeostasis, contribute to this time‐dependent functional decline [1], heterochronic parabiosis models, which involve surgically joining two animals of different ages, have highlighted the importance of circulating pro‐ageing or pro‐youthful factors in age‐related physiological changes [2, 3, 4]. Therefore, researchers around the world have been making tremendous endeavours to uncover specific blood‐borne factors present in the systemic milieu for the purpose of further understanding ageing process [5].

Sarcopenia is characterized by a gradual and generalized loss of skeletal muscle mass and function and is mainly attributable to imbalance between muscle protein synthesis and breakdown during ageing [6, 7]. This condition is closely linked to adverse outcomes, including frailty, falls and functional decline, all of which make it impossible for older people to live independently [8, 9]. Sarcopenia is thus a crucial trait of ageing and is regarded as a ‘geriatric giant’ due to the explosively increased incidence in super‐aged societies [10]. Consequently, in order to lead a healthy old life, it is essential to identify various muscle‐related contributors and to develop biomarkers and therapeutic targets for sarcopenia based on them.

Beta‐2 microglobulin (B2M) constitutes the light chain of major histocompatibility complex class I (MHC‐I) molecules, which dissociate from nucleated cells and the cell membrane in response to various stimuli, such as endoplasmic reticulum stress and inflammatory reaction [11, 12]. Apart from its traditional immunological function [13], a growing body of evidence suggests the potential involvement of B2M in age‐related degeneration as well. When compared to age‐matched young isochronic parabionts, plasma from young heterochronic parabionts exhibited increased B2M levels after exposure to aged blood [3, 14], and circulating B2M concentrations demonstrated a consistent rise with chronological age in both humans and mice [14]. Furthermore, exogenous B2M injection disrupts cognitive function and neurogenesis in young mice, but the lack of inherent B2M expression or the blockade of B2M function counteract cognitive decline associated with ageing and augments neurogenesis in aged mice [14, 15]. These findings have brought great attention to circulating B2M as a systemic factor with implications in the pro‐ageing process and have raised the possibility that B2M may also be involved in the pathogenesis of sarcopenia, a key component of ageing phenotypes. To clarify the potential role of B2M in muscle metabolism, we investigated the effects of B2M on in vitro and animal muscle biology and its clinical relevance for sarcopenia parameters in older adults.

## 2 Materials and Methods

### 2.1 Cell Culture and Reagents

Mouse C2C12 myoblasts were obtained from the American Type Culture Collection (Manassas, VA) and cultured in Dulbecco's Modified Eagle's Medium (DMEM) supplemented with 15% fetal bovine serum, 20 mM hydroxyethyl piperazine ethane sulfonic acid, 2 mM L‐glutamine, 100 U/mL penicillin and 0.1 mg/mL streptomycin (Life Technologies Corp., Carlsbad, CA). The cells were maintained at 37°C in a humidified atmosphere with 5% CO_2_. To induce myogenesis, cells were grown to 90% confluence in the maintenance medium and subsequently switched to differentiation medium (DMEM with 2% horse serum) for a duration of 3–4 days. The myogenic effects of B2M to differentiated myotube were evaluated using C2C12 cells, specifically the GFP 1‐10 Clone (Cat. No. T8003) and the GFP 11 Clone (Cat. No. T8004), both obtained from Applied Biological Materials Inc. (Richmond, bc, Canada). Recombinant B2M was procured from Lee Biosolutions (Cat. No. 126‐11‐10; Maryland Heights, MO).

### 2.2 Animals

Animal experimental procedures were approved by the Institutional Animal Care and Use Committee of the Asan Institute for Life Sciences (No. 2019‐12‐143). Three‐month‐old male C57BL/6 mice (Orient Bio, Seongnam, South Korea) were used in this study. Recombinant B2M (250 μg/100 μL) or PBS (100 μL) was intraperitoneally injected with a 31‐G needle 5 times per week for 4 weeks (6 mice per group). Mice were sacrificed at 16 weeks of age following treatment by cardiac puncture. All treatment groups were weight matched and randomized to treatment at the initiation of an experiment. The researcher conducting the treatment was not blinded to the experimental groups, but the researcher assessing muscle parameters was blinded to the analyses.

### 2.3 Study Participants for Clinical Research

This cross‐sectional study was conducted among a cohort of older adults aged 65 years or older residing in the community in South Korea. These individuals underwent a comprehensive functional assessment at the outpatient geriatric clinic of Asan Medical Center in Seoul between May 2020 and November 2020. Their visits to the clinic were primarily for the management of chronic conditions such as osteoporosis, osteoarthritis, hyperlipidaemia and hypertension or for the evaluation of common nonspecific symptoms associated with ageing, such as fatigue and loss of appetite. None of the participants were residing in hospitals or nursing homes; all of them had the capability to move around independently, either with or without walking aids. Individuals with a life expectancy of less than 1 year due to malignancy, advanced heart failure or end‐stage renal disease were not included in the study. All 158 eligible participants provided written informed consent and agreed to have their blood samples collected for enrolment in the research. The study received approval from the Institutional Review Board (IRB no. 2020‐0259) and adhered to the ethical guidelines for human experimentation outlined in the Declaration of Helsinki.

Additional information for in vitro, animal and clinical research.

Detailed information is provided in the Supporting Information.

### 2.4 Statistical Analysis

The in vitro data are presented as the mean ± standard error of the mean derived from a minimum of three independent experiments, each with triplicate measurements, unless stated otherwise. We assessed the significance of variations among three or more groups using analysis of variance with subsequent post hoc analysis using Tukey's honest significance test, and we evaluated the differences between two groups using the Mann–Whitney *U* test.

The clinical data were presented as means ± standard deviation, along with numbers and percentages. To compare the baseline characteristics of participants with and without sarcopenia, we used Student's *t*‐test for continuous variables and the chi‐square test for categorical variables. We assessed the association between age and serum B2M levels through Pearson correlation analyses. Analysis of covariance (ANCOVA) was employed to compare the estimated mean serum B2M levels with respect to sarcopenia status and related parameters while adjusting for sex, age and body mass index (BMI). Linear regression analysis was conducted to evaluate the relationship between serum B2M levels and specific muscle parameters relevant to sarcopenia, both before and after controlling for sex, age and BMI. Logistic regression analysis was performed to calculate odds ratios (ORs) for the risk of sarcopenia and adverse muscle outcomes based on serum B2M increments. All statistical analyses were carried out using SPSS Version 18.0 (SPSS Inc., Chicago, IL). A significance level of *p* < 0.05 was considered statistically significant.

## 3 Results

### 3.1 Effects of Recombinant B2M on in Vitro Myogenesis

To elucidate the role of B2M in sarcopenia, we first examined its tissue‐specific expression in skeletal muscle using publicly available datasets from human and murine models (Figure S1a). The analysis revealed an age‐dependent increase in B2M expression, indicating its potential involvement in the pathogenesis of sarcopenia. As a proof of concept, we subsequently quantified circulating B2M levels in young and aged mice. Consistent with the observed reduction in skeletal muscle mass in aged mice, serum B2M levels showed a striking 7.2‐fold increase (ranging from 7.5 to 10.0 μM) (Figure S1b). In this context, we systematically evaluated the effects of B2M on muscle metabolism.

**FIGURE 1:**
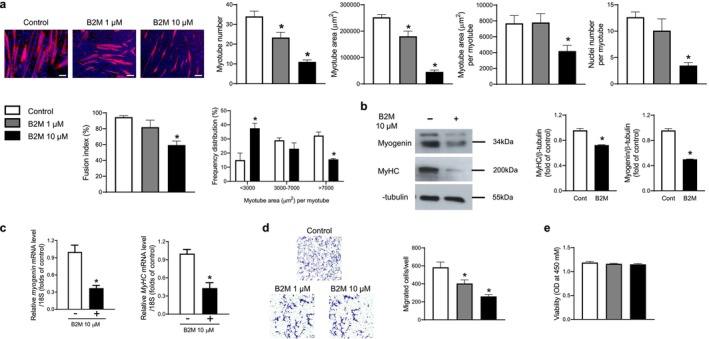
Recombinant B2M suppresses in vitro muscle differentiation. (a) Mouse C2C12 myoblasts were differentiated into myotubes with 2% horse serum after exposure to the indicated concentrations of recombinant B2M for 3 days. Myotubes were stained with anti‐MyHC antibody, whereas nuclei were counterstained with 4,6‐diamidino‐2‐phenyindole. Quantitative results per field are presented (*n* = 4). (b) Western blot and (c) quantitative reverse‐transcription polymerase chain reaction analyses of myogenin and MyHC in C2C12 cells with 2% horse serum in the presence or absence of 10 μM B2M for 3 days (*n* = 3). (d) The directional migration and (e) viability of mouse C2C12 myoblasts were assessed by a Boyden chamber system and cell counting kit‐8 assay after exposure to the indicated concentrations of recombinant B2M for 6 and 24 h, respectively (*n* = 5). Scale bars: 100 μm (a) and 50 μm (d). B2M, β2‐microglobulin; MyHC, myosin heavy chain; OD, optical density. *p < 0.05 vs. untreated control.

For the in vitro experiments, myoblasts from the C2C12 cell line were differentiated into mature myotubes with and without recombinant B2M. Myotube number, myotube area, myotube area per myotube, nuclei number per myotube and fusion index were all significantly reduced following recombinant B2M treatment in a dose‐dependent manner (Figure 1a). Furthermore, recombinant B2M treatment resulted in a decrease in the proportion of large myotube area and an increase in the proportion of small myotube area (Figure 1a). Consistently, western blot and quantitative RT‐PCR analyses revealed that recombinant B2M significantly reduced the protein and mRNA expression levels of myogenic differentiation markers, including myogenin and myosin heavy chain (MyHC) were markedly decreased by recombinant B2M (Figure 1b,c). When we looked into the potential impacts of B2M on other muscle biology, recombinant B2M significantly suppressed the migration of C2C12 myoblasts (Figure 1d) without affecting the viability (Figure 1e). These findings provide in vitro proof that B2M plays a detrimental role in muscle metabolism primarily by inhibiting muscle differentiation.

### 3.2 Increased Intracellular Reactive Oxygen Species (ROS) Production Underlies the Inhibitory Effects of B2M on Myogenesis

**FIGURE 2:**
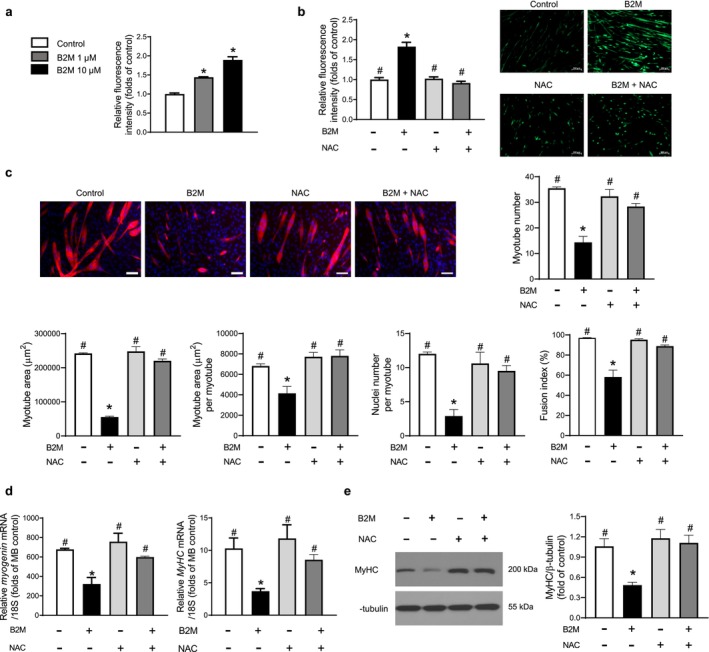
The inhibitory effects of B2M on myogenesis are mediated by increased intracellular ROS generation. (a) Mouse C2C12 myoblasts were differentiated into myotubes with 2% horse serum after exposure to the indicated concentrations of recombinant B2M for 3 days. Intracellular ROS levels were measured using a fluorescent probe, chloromethyl derivative of 2′,7′‐dichlorofluorescein diacetate (CM‐H_2_DCFDA; *n* = 5). (b,c) Mouse C2C12 myoblasts were differentiated into myotubes with 2% horse serum in the presence or absence of 10 μM B2M and/or 1 mM NAC for 3 days. (b) Intracellular ROS levels were measured using H_2_DCFDA (*n* = 3). (c) Myotubes were stained with anti‐MyHC antibody, whereas nuclei were counterstained with 4,6‐diamidino‐2‐phenyindole. Quantitative results per field are presented (*n* = 3). (d) Quantitative reverse‐transcription polymerase chain reaction and (e) western blot analyses of MyHC and/or myogenin in C2C12 cells with 2% horse serum in the presence or absence of 10 μM B2M and/or 1 mM NAC for 3 days (*n* = 3). Scale bars: 100 μm (b) and 100 μm (c). B2M, β2‐microglobulin; MyHC, myosin heavy chain; NAC, N‐acetyl cysteine; ROS, reactive oxygen species. *p < 0.05 vs. untreated control; #*p* < 0.05 vs. 10 μM B2M.

To ascertain the extent of intracellular reactive oxygen species (ROS) production, we assessed chloromethyl derivative of 2′,7′‐dichlorofluorescein diacetate (CM‐H2DCFDA) intensity in the presence or absence of recombinant B2M treatment during myogenesis. The intracellular ROS levels in myotubes were enhanced by B2M in a dose‐dependent manner with a 1.9‐fold rise in CM‐H2DCFDA content at 10 μM B2M (Figure 2a). It was interesting to note that pretreatment with N‐acetyl cysteine (NAC), a strong biological thiol antioxidant, dramatically decreased the stimulation of intracellular ROS production caused by B2M in myotubes (Figure 2b). NAC also significantly attenuated the effects of B2M on myotube number, myotube area, myotube area per myotube, nucleus number per myotube and fusion index (Figure 2c). Consistently, decreased mRNA and protein expressions of myogenin and/or MyHC by B2M were markedly reversed by NAC pretreatment during myogenesis (Figure 2d,e). These data demonstrated the significance of oxidative stress in the suppression of muscle differentiation brought on by treatment with recombinant B2M.

### 3.3 B2M Induces Differentiated Myotube Atrophy via ROS‐Mediated ITGB1 Deficiency and Subsequent Suppression of the FAK/AKT/ERK/FoxO Axis

**FIGURE 3:**
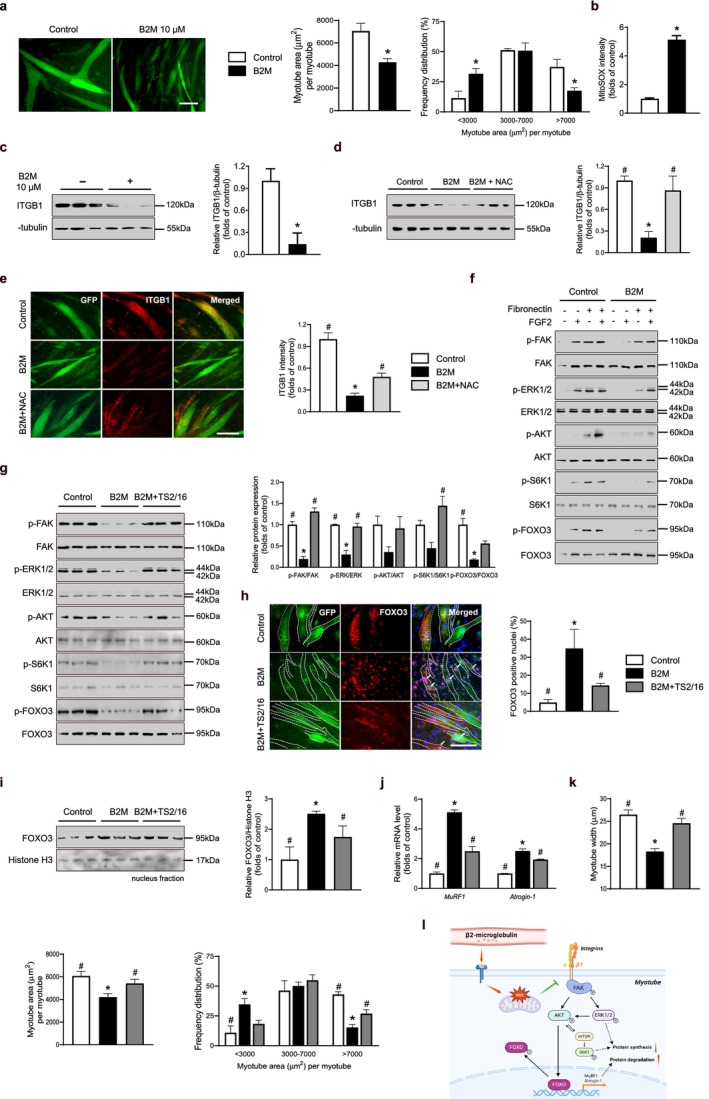
B2M induces differentiated myotube atrophy via ROS‐mediated ITGB1 deficiency. (a) Recombinant B2M induced differentiated myotube atrophy. Mouse C2C12 myoblasts were differentiated into myotubes with 2% horse serum for 4 days, followed by treatment with either a vehicle control or recombinant 10 μM B2M for 24 h. Quantitative results per field are presented (*n* = 3). (b) ROS levels were measured using MitoSOX kit (*n* = 5). (c,d) Western blot analyses of ITGB1 in differentiated myotubes in the presence or absence of 10 μM B2M (c) and/or 1 mM NAC (d) (*n* = 3). (e) Representative immunofluorescence images of ITGB1 (Red) expression in differentiated myotubes. Quantitative results per field are presented (*n* = 5). (f) Western blot analyses of downstream signalling cascades of ITGB1 in myotubes treated with vehicle or 10 μM B2M, in the presence or absence of 10 μg/mL fibronectin and 50 ng/mL FGF2. (g) Western blot analyses of downstream signalling cascades of ITGB1 in myotubes treated with 10 μM B2M and/or 10 μg/mL TS2/16 in the presence of fibronectin and FGF2 (*n* = 3). (h) Representative immunofluorescence images of FoxO nuclear translocation in differentiated myotubes. Quantitative results per field are presented (*n* = 3). (i) Western blot analyses of nuclear FoxO in extracted nuclei (*n* = 3). (j) Quantitative reverse‐transcription polymerase chain reaction of MuRF1 and Atrogin‐1 (*n* = 3). (k) Parameters of myotubes treated with 10 μM B2M and/or 10 μg/mL TS2/16. (l) Schematic illustration of potential mechanism underlying B2M‐induced myotube atrophy. Scale bars: 100 μm (a,e,h). B2M, β2‐microglobulin; FGF2, fibroblast growth factor 2; FoxO, forkhead box O; ITGB1, integrin β1; NAC, N‐acetyl cysteine; ROS, reactive oxygen species. *p < 0.05 vs. untreated control; #*p* < 0.05 vs. 10 μM B2M.

To ascertain the effects of B2M in differentiated myotubes, myoblasts were induced to differentiate for 4 days, followed by treatment with either a vehicle control or recombinant B2M for 24 h. Myotubes treated with B2M exhibited pronounced atrophy, characterized by a significant reduction in average myotube area and a marked decrease in the proportion of large myotubes (Figure 3a). Notably, B2M treatment led to an increase in intracellular ROS levels in differentiated myotubes (Figure 3b), implicating oxidative stress as a potent mediator of B2M‐induced atrophy as well. To investigate the underlying molecular mechanisms, we analysed the expression of integrin β1 (ITGB1), a pivotal regulator of cell–extracellular matrix (ECM) interactions and mechanotransduction, which plays a critical role in skeletal muscle regeneration [16]. Consistent with previous findings that ROS suppresses ITGB1 expression in epithelial cells [17], we observed a significant downregulation of ITGB1 in response to recombinant B2M treatment (Figure 3c). Importantly, co‐treatment with the antioxidant NAC restored ITGB1 expression (Figure 3d), highlighting the central role of B2M‐induced oxidative stress in the suppression of ITGB1. Immunofluorescence analysis further supported these findings, revealing that recombinant B2M contributed to ITGB1 expression deficiency and induced myotube atrophy; however, these effects were substantially ameliorated by supplementation with NAC (Figure 3e).

To further delineate the downstream pathways regulated by B2M, we treated myotubes with fibronectin, an ECM molecule integral to integrin‐receptor tyrosine kinase crosstalk, and/or FGF2, a potent growth factor that synergizes with integrins to regulate cell signalling and differentiation [16, 18]. Fibronectin and FGF2 co‐treatment robustly activated downstream effectors, including FAK, AKT, S6K1 and ERK, in normal myotubes (Figure 3f), which are well‐documented mediators of skeletal muscle regeneration [19]. In contrast, myotubes rendered ITGB1‐deficient by B2M exhibited defective activation of these signalling pathways. To determine the potential mechanistic link, we treated B2M‐injured myotubes with the ITGB1‐activating antibody TS2/16 [16], in conjunction with FGF2 and fibronectin. TS2/16 evidently rescued the B2M‐suppressed downstream signalling cascade (Figure 3g), highlighting the regulatory role of B2M in muscle homeostasis by targeting ITGB1. Given the well‐characterized role of AKT in phosphorylating Forkhead box O (FoxO) transcription factors—key determinants of muscle atrophy via the transcriptional activation of E3 ubiquitin ligases such as MuRF‐1 and Atrogin‐1 [20]—we further examined FoxO phosphorylation and intracellular localization. Recombinant B2M significantly enhanced FoxO nuclear translocation (Figure 3h), as evidenced by the increased nuclear accumulation of FoxO in myotubes (Figure 3i and Figure S2), facilitating the transcription of atrophy‐related factors (Figure 3j). Conversely, TS2/16 restored FoxO phosphorylation through activation of the FAK/AKT pathway, thereby ameliorating myotube atrophy (Figure 3k). Collectively, these data demonstrate the significant impact of B2M on myotube atrophy via ROS‐mediated ITGB1 downregulation, leading to impaired activation of the FAK/AKT/ERK signalling cascade and enhanced nuclear translocation of FoxO transcription factors (Figure 3l).

### 3.4 Decrease Muscle Size, Muscle Strength and Physical Performance by Systemic B2M Treatment in Mice

**FIGURE 4:**
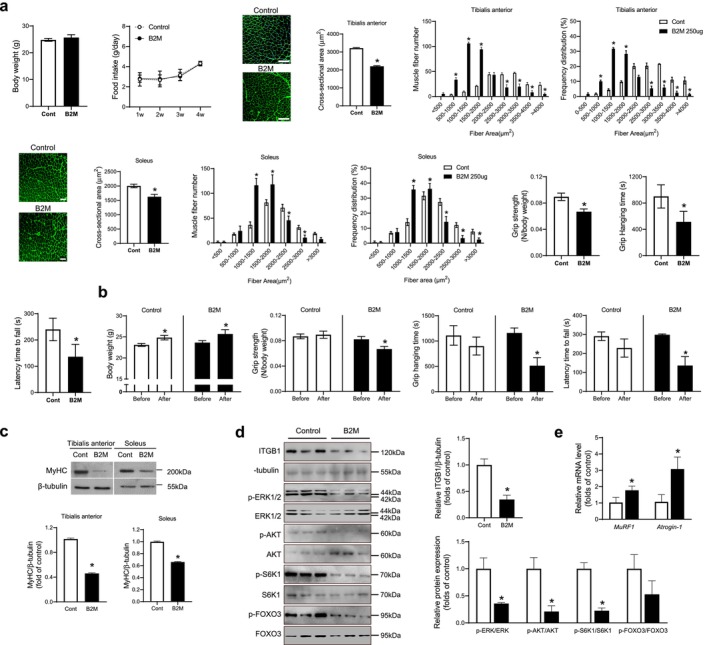
Systemic B2M treatment reduces muscle strength as well as the size of muscle fibre in mice. Three‐month‐old male mice were intraperitoneally injected with PBS (100 μL) or recombinant B2M (250 μg/100 μL) for 4 weeks (*n* = 6 per group). (a) Muscle phenotypes were compared between untreated controls and B2M‐injected mice after 4 weeks. Body weight, food intake, tibialis anterior size, soleus size, grip strength, grid hanging time and latency time to fall from the rotating rod. (b) Muscle phenotypes were compared before and after 4 weeks of treatment in each of the control mice and B2M‐injected mice. Body weight, grip strength, grid hanging time and latency time to fall from the rotating rod. (c) Western blot analyses of MyHC in tibialis anterior and soleus muscles after 4 weeks of treatment. (d,e) Western blot analyses of ITGB1 and its downstream signals (d) and quantitative reverse‐transcription polymerase chain reaction analyses of atrophy‐related factors (e) in tibialis anterior muscles from untreated controls or B2M‐injected mice. Scale bars: 100 μm (a). B2M, β2‐microglobulin; ITGB1, integrin β1; MyHC, myosin heavy chain; PBS, phosphate‐buffered saline. *p < 0.05 vs. untreated control or before treatment.

To investigate the in vivo effects of B2M on muscle phenotypes, 3‐month‐old male mice were intraperitoneally injected with 250 μg of B2M for 4 weeks. Blood B2M levels exhibited a 5.9‐fold increase following administration, reaching approximately 12 μM (Figure S3). Untreated controls and B2M‐injected mice were matched for body weight at baseline, and there was no difference in body weight and food intake after 4 weeks of treatment between groups as well (Figure 4a). Compared to the controls, B2M treatment reduced the cross‐sectional area (CSA) of TA and soleus muscles by 31.4% and 18.8%, respectively, and increased the number of small fibres while decreasing the number of large fibres in both muscle types (Figure 4a). Immunostaining of both TA and soleus muscles revealed that B2M treatment did not alter the proportions of Type 1, Type 2a or Type 2b fibres, indicating that B2M does not induce muscle fibre type switching but rather causes muscle atrophy (Figure S4). Furthermore, the grip strength, grid hanging time and latency time to fall from the rotating rod in mice treated with B2M decreased by 25.3%, 43.0% and 43.3%, respectively, compared to untreated controls (Figure 4a). Next, changes in muscle functions were evaluated before and after 4 weeks of treatment in each of the control mice and B2M‐injected mice. In both groups, body weight significantly increased to a similar extent after 4 weeks (Figure 4b). However, grip strength, grid hanging time and latency time to fall from the rotating rod decreased by 18.8%, 55.7% and 54.4%, respectively, after 4 weeks of B2M treatment, whereas there was no change in the untreated controls (Figure 4b). Consistent with these results, western blot analyses of TA and soleus muscles showed that B2M treatment markedly inhibited the expression of MyHC, a marker of terminal differentiation (Figure 4c).

To validate the proposed regulatory mechanisms of B2M suggested by in vitro experiments, we analysed TA muscle from B2M‐treated mice using western blotting. The results revealed a reduction in ITGB1 expression, accompanied by the suppression of its downstream signalling cascades (Figure 4d) and the transcriptional activation of atrophy‐related factors (Figure 4e).

### 3.5 B2M Administration Altered Genes Associated With Muscle Metabolism, Tissue Remodelling and Mitochondrial Homeostasis

**FIGURE 5:**
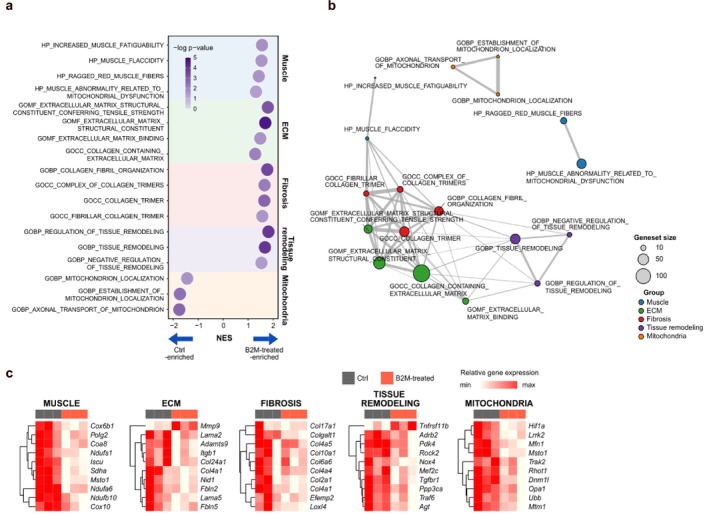
Muscle atrophy induced by B2M is attributable to regulation of gene sets of muscle metabolism, tissue remodelling and mitochondrial homeostasis. (a) Bubble plots summarizing the significantly altered gene sets as results of gene set enrichment analysis (GSEA). (b) Gene Ontology (GO) enrichment analysis demonstrating dysregulation of gene sets in muscles from B2M‐injected mice. (c) A heatmap showing differential gene expressions between two groups, which correlates with muscle metabolism, tissue remodelling and mitochondrial homeostasis. B2M, β2‐microglobulin.

To gain deeper insights into the mechanisms underlying B2M‐mediated muscle phenotypes, we performed transcriptome profiling using gene set enrichment analysis, an unbiased method (Figure 5a). The B2M group exhibited positive normalized enrichment scores (NES) in gene sets associated with disrupted muscle metabolism (shaded in blue), excessive ECM and muscle remodelling (shaded in green and purple) and pathological ECM deposition (shaded in pink). Notably, gene sets related to mitochondrial dynamics (shaded in orange) were enriched with negative NES in the B2M group, whereas those related to oxidative stress (shaded in yellow) were enriched with positive NES (Figure S5), suggesting that B2M modulates mitochondrial homeostasis. Gene Ontology enrichment analysis further confirmed profound dysregulation in B2M‐impacted muscles, highlighting impaired mitochondrial dynamics, functional deficits and disrupted muscle regeneration, indicative of pathological remodelling (Figure 5b). These findings were supported by gene expression heatmaps, which revealed distinct alterations in key genes across multiple categories (Figure 5c).

Building on these findings, we further investigated the effects of B2M on mitochondrial metabolism to elucidate its role in muscle metabolism. B2M inhibited the transcript expression of *Ppargc1a* and *Tfam* both in vivo and in vitro (Figure S6a,b) and suppressed mitochondrial function in vitro (Figure S6c,d). However, these declines were rescued by the antioxidant NAC in vitro, suggesting that B2M‐induced mitochondrial dysregulation is linked to ROS production. We further conducted an AI‐powered, label‐free three‐dimensional live imaging analysis to assess mitochondrial dynamics. Recombinant B2M impaired mitochondrial biogenesis (Figure S6e,f) and facilitated mitochondrial fragmentation, as evidenced by reductions in mitochondrial morphological parameters (Figure S6g,h) and an increased proportion of fragmented mitochondria (Figure S6i). These findings underscore the detrimental role of ROS in mitochondrial integrity [21], which was reversed by NAC, highlighting the ROS‐mediated regulation of mitochondrial metabolism by B2M.

### 3.6 Differences in Serum B2M Level According to the Status of Sarcopenia and Muscle Phenotypes in Older Adults

To ascertain the clinical relevance of B2M's effects on muscle homeostasis in in vitro and animal experiments, circulating B2M concentrations were measured in 158 older adults aged 65 and older and their baseline characteristics are presented in Table S1. Among the 118 controls without sarcopenia and 40 cases with sarcopenia, 97 (82.2%) and 29 (72.5%) were women, respectively. The mean age of controls was 75.3 ± 5.2 years, whereas that of cases was 79.7 ± 4.6 years (*p* < 0.001). Compared to the control group, participants with sarcopenia exhibited lower weight, BMI, ASM, SMI, grip strength, gait speed and SPPB total score (*p* = 0.023 to <0.001). On the other hand, their chair stand test time and sarcopenia phenotype score (SPS) score were significantly higher (*p* = 0.003 and < 0.001, respectively).

Pearson correlation analyses with scatter plots revealed that serum B2M concentrations increased with age, which in consistent with previous reports (γ = 0.446, *p* < 0.001; Figure S7) [22].

**FIGURE 6:**
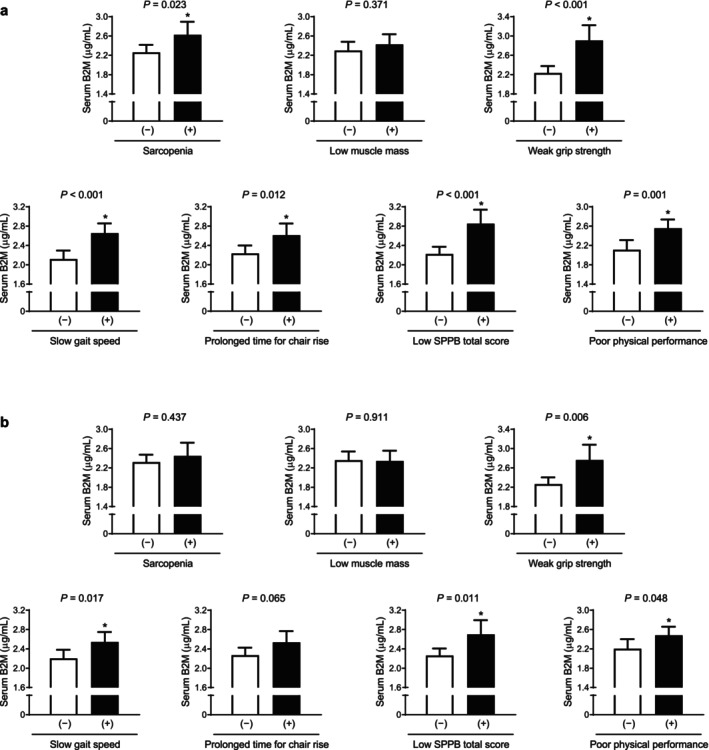
Serum B2M level by sarcopenia and muscle‐related parameters before (a) and after (b) adjusting for age, sex and body mass index. The estimated mean values with 95% confidence intervals were calculated and compared using analysis of covariance. * indicates a statistically significant difference from control. B2M, β2‐microglobulin.

Differences in serum B2M levels according to status of sarcopenia and related specific component were evaluated using ANCOVA before and after adjustment for confounding factors. In a crude analysis, serum B2M levels were higher in participants with sarcopenia, weak grip strength, slow gait speed, prolonged chair stand test time, low SPPB total score and poor physical performance (*p* = 0.023 to < 0.001), although there was no significant difference of serum B2M between those with and without low muscle mass (Figure 6a). After adjustment for sex, age and BMI, the statistical significance of serum B2M levels in relation to the status of weak grip strength, slow gait speed, low SPPB total score and poor physical performance persisted (*p* = 0.006 to 0.048; Figure 6b). However, in this multivariable adjustment model, serum B2M concentrations were no longer different between those with and without sarcopenia or prolonged chair stand test time.

### 3.7 Association Between Serum B2M Level and Sarcopenia‐Related Parameters in Older Adults

Linear regression analyses were conducted to explore the relationship of serum B2M levels with specific muscle parameters relevant to sarcopenia (Table 1). Regardless of adjustment models, circulating B2M levels were positively associated with SPS and inversely associated with grip strength, gait speed and SPPB total score (*p* < 0.001–0.024), but not with SMI and chair stand test time.

**TABLE 1:** Associations of serum B2M level with muscle‐related parameters by linear regression analysis.

Dependent variable	Unadjusted		Age, sex and BMI adjusted	
	B	*p*	B	*p*
Sarcopenia phenotype score	**0.300**	**< 0.001**	**0.178**	**0.024**
Skeletal muscle mass index	−0.013	0.868	0.053	0.396
Grip strength	**−0.291**	**< 0.001**	**−0.216**	**0.001**
Usual gait speed	**−0.364**	**< 0.001**	**−0.249**	**0.002**
Chair rise test time	0.154	0.054	0.060	0.472
SPPB total score	**−0.316**	**< 0.001**	**−0.212**	**0.008**

*Note:* The Enter method is applied to this model with serum B2M level as an independent variable. Bold numbers indicate statistically significant values.

Abbreviations: B, standardized regression coefficient; B2M, β2‐microglobulin; SPPB, short physical performance battery.

The risk of sarcopenia and poor muscle outcomes according to serum B2M levels in older adults was assessed by logistic regression analyses (Table 2). Prior to adjustment for confounding variables, the ORs for weak grip strength, slow gait speed, low SPPB total score and poor physical performance per 1 μg/mL increase in serum B2M level were 2.23, 2.18, 2.16 and 1.95, respectively (*p* < 0.001–0.002), and increased risk of these conditions with elevated serum B2M levels was still significant even after accounting for age, sex and BMI (*p* = 0.019–0.046). However, higher ORs for sarcopenia and prolonged chair stand test time by serum B2M increase were only evident in the univariate model (*p* = 0.027 and 0.015, respectively), with statistical significance diminishing upon adjustment for potential confounding factors. Furthermore, the risk of low muscle mass per 1 μg/mL increase in serum B2M level was insignificant in either unadjusted or adjusted analyses.

**TABLE 2:** Association of presence of sarcopenia or abnormalities in sarcopenia parameters with serum B2M level by logistic regression analysis.

Dependent variable	Unadjusted		Age, sex and BMI adjusted	
	OR (95% CI) per serum B2M increment	*p*	OR (95% CI) per serum B2M increment	*p*
Sarcopenia	**1.57 (1.05–2.35)**	**0.027**	1.22 (0.78–1.93)	0.382
Low muscle mass	1.18 (0.82–1.69)	0.370	1.03 (0.66–1.16)	0.898
Weak grip strength	**2.23 (1.40–3.55)**	**0.001**	**1.85 (1.11–3.10)**	**0.019**
Slow gait speed	**2.18 (1.42–3.36)**	**<0.001**	**1.72 (1.08–2.72)**	**0.021**
Prolonged time for chair rise	**1.62 (1.10–2.40)**	**0.015**	1.47 (0.96–2.23)	0.075
Low SPPB total score	**2.16 (1.38–3.37)**	**0.001**	**1.74 (1.06–2.85)**	**0.029**
Poor physical performance	**1.95 (1.27–2.99)**	**0.002**	**1.60 (1.01–2.52)**	**0.046**

*Note:* Bold numbers indicate statistically significant values.

Abbreviations: B2M, β2‐microglobulin;CI, confidence interval; OR, odds ratio; SPPB, short physical performance battery.

## 4 Discussion

Given the backgrounds implicating B2M in various age‐related diseases, we conducted in vitro and animal research, which demonstrated that recombinant B2M treatment suppresses myogenesis and induces muscle atrophy by triggering the generation of intracellular ROS. Moreover, our subsequent investigation to understand its clinical implications revealed that higher serum B2M levels were associated with a higher risk of weak grip strength, slow gait speed, a low SPPB total score and poor physical performance in older adults. These results provide experimental evidence that B2M, which exerts detrimental effects on muscle metabolism, could be a potential therapeutic target for sarcopenia. Additionally, they suggest that circulating B2M could serve as one of the blood‐based biomarkers for assessing poor muscle health in humans.

Sarcopenia, characterized by the loss of muscle mass and function, is becoming increasingly important from a public health perspective in the era of extreme ageing, as it is associated with various adverse outcomes in the elderly population [8, 22]. Despite its growing significance, there is currently no approved treatment for this condition. This underscores the fact that sarcopenia remains a hot topic in ageing research, highlighting the urgent need for innovative approaches to address this complex syndrome and improve the quality of life for older individuals. As part of our efforts to uncover potential factors contributing to the development of sarcopenia, we have been particularly focused on exploring the role of B2M in muscle metabolism. B2M is a non‐glycosylated protein consisting of 119 amino acid residues, with a secreted form comprising 99 amino acids and a molecular weight of 12 kDa [23]. The circulating B2M concentration consistently showed elevation not only in young partners within heterochronic parabiosis experiments and aged mice but also in older humans [3, 14], and our clinical study, which included older adults, confirmed a positive correlation between age and serum B2M level (Figure S7). Importantly, the administration of recombinant B2M resulted in impaired neurogenesis, synaptic dysfunction and memory deficits in mice, whereas the use of B2M antagonists or genetic ablation of B2M effectively mitigated these detrimental phenotypes in aged mice [14, 15]. These discoveries have garnered significant interest in circulating B2M as a systemic factor with relevance to the ageing process. Given that sarcopenia is a key phenotype of ageing, we conducted current experiments and found that recombinant B2M inhibited in vitro myogenesis, induced myotube atrophy and decreased muscle size, muscle strength and physical performance in mice. Collectively, manipulations mitigating the effects of various pro‐ageing factors have been proposed as potentially effective approaches for slowing or reversing the ageing process and B2M may be one of these therapeutic targets for age‐related diseases, including sarcopenia and dementia.

What sets this study apart from previous research is that we conduced translation research to elucidate the role of B2M in human muscle health by expanding upon the findings from an experimental study that confirmed its detrimental impact on muscle metabolism. Due to the impossibility of conducting interventional trials in humans without prior safety verification from preclinical data, the roles of candidate factors in humans must be extrapolated from observational results, which has led us to undertake current clinical study. Consequently, in line with results from in vitro and animal experiments, high serum B2M concentrations were associated with poor muscle phenotypes in older adults. A particularly intriguing finding in our clinical study is that serum B2M levels were primarily associated with muscle strength and physical performance, showing limited correlation with muscle mass, among the diagnostic criteria for sarcopenia. Indeed, muscle mass, muscle strength and physical performance each offer unique insights into different aspects of muscle health. Muscle mass primarily reflects the quantity or size of muscle tissue in the body, signifying its structural composition [24]. Muscle strength represents how much force a muscle or group of muscles can generate during contraction [25], whereas physical performance encompasses a range of abilities such as coordination, endurance and power, reflecting the integrated efficiency of muscle groups during activities [26]. Importantly, various factors, such as neural control, coordination and the quality of muscle tissue, can impact muscle strength and physical performance independently of muscle mass [27, 28, 29]. Therefore, we speculate that B2M may directly affect the functional aspects of muscles, such as their contractile properties, neuromuscular efficiency or energy utilization, rather than causing a significant change in muscle size in humans. All these findings emphasize the complexity and multifaceted nature of muscular physiology.

In order to explore the potential mechanisms that underlie the adverse impact of B2M on muscle metabolism, our research has centred on examining alterations in redox signalling. Within a well‐functioning skeletal muscle system, the generation of oxidative species is carefully balanced by their elimination through both exogenous and endogenous antioxidant molecules [30, 31]. Nonetheless, an imbalance between these processes under specific pathological conditions disrupts the usual redox equilibrium, a phenomenon commonly described as ‘oxidative stress’ [32]. Several research findings have consistently suggested that oxidative stress can result in a range of detrimental effects within skeletal muscle. These effects encompass a triggered inflammatory response, excitation–contraction uncoupling, an increase in myonuclear apoptosis, dysregulated autophagy, heightened ubiquitin proteasome activity, disruptions in mineral homeostasis and mitochondrial dysfunction [30, 33, 34]. These multifaceted processes collectively contribute to muscle fatigue, impaired recovery after exercise or injury and muscle atrophy due to increased protein breakdown, ultimately compromising the strength, endurance and overall performance of skeletal muscle. Notably, experimental research has revealed that B2M induces cellular damage by augmenting the ROS production [35]. Moreover, a correlation between B2M and oxidative stress has been observed in the elderly population [36], suggesting that oxidative stress could potentially mediate the role of B2M as a pro‐ageing factor. Building upon this background, our study delved into the investigation of whether recombinant B2M leads to an increase in intracellular ROS production within muscle cells and whether antioxidants reverse the detrimental impact of B2M on myogenesis and myotube atrophy. As a result, our findings underscore the pivotal role of oxidative stress as a key regulator in mediating the influence of B2M on muscle metabolism.

This study presents a significant advantage as it incorporates comprehensive investigations at the cellular, animal and clinical levels, collectively providing robust evidence to elucidate the influence of B2M on muscle metabolism. The other major strength is our clinical assessment of all required parameters to define sarcopenia, including muscle mass, handgrip strength, gait speed, SPPB score and five‐time chair‐stand test, thereby enhancing the reliability of our findings [8, 37]. Furthermore, we utilized Asian‐specific cut‐off values for the diagnosis of sarcopenia in older adults, recognizing that muscle phenotypes can exhibit variations based on lifestyles, ethnicities, cultural backgrounds and body sizes [37].

In conclusion, recombinant B2M inhibited in vitro myogenesis, induced myotube atrophy and reduced muscle size, strength and physical performance in mice. Furthermore, higher serum B2M levels, which increased with age, were significantly associated with poor muscle phenotypes in older adults. These consistent experimental and clinical findings support the detrimental impact of B2M on muscle homeostasis, suggesting that B2M may be a therapeutic target for sarcopenia and raising the possibility that measuring serum B2M levels could offer additional insights for evaluating muscle health in old age. Further meticulously designed large‐scale longitudinal studies are imperative to confirm the role of circulating B2M as a blood‐based biomarker for predicting the risk of sarcopenia.

## Conflicts of Interest

The authors declare no conflicts of interest.

## Supporting information


**Data S1** Supporting Information.

**Figure S1** B2M levels increase with ageing. (a) B2M levels exhibit an age‐dependent increase in skeletal muscle from both human and murine models. (b) Serum B2M levels increase in aged mice. B2M, β2‐Microglobulin. *p < 0.05 vs. young mice.
**Figure S2.** Western blot analyses of β‐tubulin and Histone H3 in nucleus and cytosol. B2M, β2‐microglobulin; TS2/16, ITGB1‐activating antibody.
**Figure S3.** Serum B2M levels exhibited a 5.9‐fold increase following systemic B2M in mice. Three‐month‐old male mice were intraperitoneally injected with PBS (100 μL) or recombinant B2M (250 μg/100 μL) for 4 weeks (*n* = 6 per group). B2M, β2‐microglobulin; PBS, phosphate‐buffered saline. *p < 0.05 vs. untreated control or before treatment.
**Figure S4.** B2M treatment does not alter the proportions of Type I, Type IIa and Type IIb fibres in the tibialis anterior and soleus muscles. Three‐month‐old male mice were intraperitoneally injected with PBS (100 μL) or recombinant B2M (250 μg/100 μL) for 4 weeks (*n* = 6 per group). Representative images of immunofluorescent staining for laminin (green) and MyHC I, MyHC IIa and MyHC IIb (red) are shown. The relative frequency of each fibre type in the tibialis anterior and soleus muscles was evaluated. Scale bars: 100 μm. B2M, β2‐microglobulin; MyHC, myosin heavy chain; PBS, phosphate‐buffered saline.
**Figure S5.** Bubble plots summarizing B2M‐induced gene sets alteration identified by gene set enrichment analysis. B2M, β2‐microglobulin.
**Figure S6.** B2M impairs mitochondrial metabolism via ROS production. (a,b) Quantitative reverse‐transcription polymerase chain reaction of Ppargc1a and Tfam in vivo (a) and in vitro (b) (*n* = 3). (c,d) Mitochondrial function analysis via oxygen consumption rate in differentiated myotube, treated with or without B2M and/or NAC. Ant, antimycin; FCCP, carbonyl cyanide‐p‐trifluoromethoxyphenylhydrazone; Oligo, oligomycin; Rot, rotenone. (e–i) Quantitative characteristics of mitochondria in differentiated myotube, treated with or without 10 μM B2M and/or 1 mM NAC. Parameters analysed include mitochondrial number (e), volume (f), skeleton length (g) and surface area (h). Mitochondrial dynamics were assessed by calculating the percentage of mitochondria (i) exhibiting morphological features based on skeleton length: elongation (≥ 8 μm), intermediate (3–8 μm), fragmentation (< 3 μm) (*n* = 8). B2M, β2‐microglobulin; OCR, oxygen consumption rate. *p < 0.05 vs. untreated control; #*p* < 0.05 vs. 10 μM B2M.
**Figure S7.** Pearson correlation coefficient with scatter plots for the association of age with serum B2M level. * indicates a statistically significant value. B2M, β2‐microglobulin.
**Table S1.** Basic clinical characteristics of the study participants.
