# Supplementary material for: Detrimental Effects of β2‐Microglobulin on Muscle Metabolism: Evidence From In Vitro, Animal and Human Research
Source: J Cachexia Sarcopenia Muscle. 2025 Mar 3;16(2):e13745. doi: 10.1002/jcsm.13745 (PMC11873540; doi:10.1002/jcsm.13745)
Supplement: Supplementary file 1 — Data S1 Supporting Information. [file JCSM-16-e13745-s001.docx]

**Supplemental Materials and Methods**

*Immunofluorescence*

Differentiated C2C12 cells were first fixed with 4% paraformaldehyde (PFA) for 15 min, followed by two washes with phosphate-buffered saline (PBS). Subsequently, they were permeabilized in a solution containing 0.01 M sodium citrate buffer and 0.1% Triton X-100 for 10 min, and then washed twice with PBS. The cells were blocked for 1 h with 2% bovine serum albumin (BSA) in PBS and then incubated overnight at 4°C with an anti-myosin heavy chain (MyHC) antibody (MF20; Developmental Studies Hybridoma Bank, Iowa City, IA). After this primary antibody incubation, the cells were treated with Alexa Fluor 555-conjugated secondary antibodies (diluted 1:1000; Cell Signaling) for 1 h and washed with PBST (PBS containing 0.2% Tween-20). Following that, the cells were stained with 4,6-diamidino-2-phenylindole (DAPI, diluted 1:10,000; Sigma-Aldrich, St. Louis, MO) for 2 min and washed with PBS. The prepared samples were then mounted using Fluoromount G (Southern Biotech, Birmingham, AL), and fluorescence images were captured using a Carl Zeiss fluorescence microscope (Jena, Germany). Myotubes were identified as MyHC-positive cells with three or more nuclei within a continuous cytoplasm. The area occupied by MyHC-stained myotubes was quantified using ZEN 2 (blue edition) software (Carl Zeiss). The fusion index (%) was determined using the following formula: 100 × (number of nuclei in MyHC^+^ myotubes) divided by the total number of nuclei in MyHC^+^ myocytes and myotubes [S1].

To visualize ITGB1 and FOXO expression in differentiated myotubes, myotubes were incubated overnight at 4°C with primary antibodies against Integrin beta 1 (14-0299-82; Invitrogen, MA, USA) and FOXO (OSF00019W; Osenses, AU), respectively, followed by 1-hour co-incubation with an Alexa Fluor 555-conjugated secondary antibody (1:1000; Cell Signaling Technology). Fluorescence images were captured using Cytation 5 confocal microscope system (Agilent) and quantified via ImageJ software.

*Western blot analysis*

Cell lysis was performed utilizing RIPA buffer (composed of 50 mM Tris-HCl [pH 7.4], 150 mM NaCl, 1% Triton X-100, 1 mM EDTA, 1 mM EGTA, 0.1% SDS, 1% sodium deoxycholate, 1 mM Na_3_VO_4_, 1 mM NaF, 1 mM PMSF, and a protease inhibitor cocktail). Following a 30-min incubation on ice, the lysates were subjected to centrifugation at 14,000 rpm for 20 min at 4°C. The protein concentration was quantified using a BCA protein assay kit (Pierce Chemical Co., Rockford, IL). Protein samples were separated through sodium dodecyl sulfate-polyacrylamide gel electrophoresis, transferred onto polyvinylidene fluoride membranes, and subsequently subjected to immunoblotting using the following antibodies: MyHC (MF20), myogenin (sc-12732; Santa Cruz Biotechnology, Dallas, TX), ITGB1 (14-0299-82), β-tubulin ([T8328](https://www.sigmaaldrich.com/KR/en/product/sigma/t8328), Sigma-Aldrich), p-ERK1/2 (SAB4301578, Sigma-Aldrich), ERK1/2 (SAB1305560, Sigma-Aldrich), p-AKT (SAB5703038, Sigma-Aldrich), AKT (SAB4500797, Sigma-Aldrich), p-FAK (700255, Invitrogen), FAK (AHO1272, Invitrogen), p-S6K1 (MA5-15202, Invitrogen), S6K1 (9202, Cell Signaling), p-FOXO (9461, Cell Signaling), FOXO (OSF00019W), and Histone H3 (ab1791, Abcam).

*Quantitative reverse-transcription polymerase chain reaction*

Total RNA was extracted following the manufacturer's protocol using TRIzol reagent (Invitrogen, Carlsbad, CA). Subsequently, the first-strand cDNA synthesis was carried out with the Superscript III First-Strand Synthesis System (Invitrogen), employing oligo dT primers. Quantitative reverse-transcription polymerase chain reaction (qRT-PCR) was conducted in triplicate using Light Cycler® 480 SYBR Green I Master (Roche, Mannheim, Germany). The primers for *myogenin* (NM_031189.2), *MyHC* (NM_001013397.2), *Sdha* (NM_023281.1), *Ppargc1a* (NM_008904.3), *Tfam* (NM_009360.4), *Murf1* (NM_001369245.1), and *Atrogin-1* (NM_026346.3) were all sourced from Applied Biosystems (Foster City, CA). The threshold cycle (Ct) value for each target gene was normalized to the Ct value of 18S rRNA (NR_003278.3).

*Migration assay*

The chemotaxis assay was conducted utilizing a Boyden chamber system, employing transwells with an 8-μm-pore size polycarbonate membrane (Corning, NY). Cells were seeded into the inner chamber at a density of 8 × 10^4^ cells per transwell, with DMEM containing 0.2% FBS, and subsequently exposed to recombinant B2M in the outer chamber for a duration of 5 h. The cells located on the inner membrane were then thoroughly removed by gently wiping with a cotton swab. Meanwhile, the cells situated on the lower surface of the membrane were fixed using 4% PFA and subsequently stained with crystal violet. To quantify the number of C2C12 cells, images of the stained C2C12 cells were captured using cellSens Standard BX53 software (Olympus, Tokyo, Japan) and analyzed using Image J software (NIH, Bethesda, MD).

*Viability assay*

Cell viability was assessed using the Cell Counting Kit-8 (CCK-8; Dojindo, Kumamoto, Japan) as per the manufacturer's instructions. In brief, 10 μL of WST-8 dye [2-(2-methoxy-4-nitrophenyl)-3-(4-nitrophenyl)-5-(2,4-disulfophenyl)-2H-tetrazolium, monosodium salt] was introduced into each well of a 96-well plate. The mixture was incubated for 1 h, and absorbance readings were subsequently taken at 450 nm with a reference wavelength of 650 nm using a microplate reader (SPECTRAmax 340PC; Molecular Devices, Palo Alto, CA).

*Measurement of intracellular reactive oxygen species levels*

Intracellular ROS levels were quantified by employing the chloromethyl derivative of 2′,7′-dichlorofluorescein diacetate (CM-H_2_DCFDA, C6827; Invitrogen). Initially, cells were rinsed with serum-free DMEM and then incubated with 10 μM CM-H_2_DCFDA at 37°C for 30 min, shielded from light, in a 5% CO2 environment. Following this incubation, cells were washed with PBS and visualized under a fluorescent microscope (Carl Zeiss, Jena, Germany). Fluorescence intensity was also measured using a microplate reader (Infinite 200PRO; Tecan Life Sciences, Zürich, Switzerland) at an excitation wavelength of 490 nm and an emission wavelength of 520 nm.

*Nuclei extraction from differentiated myoblast*

Myoblasts were lysed in ice-cold cytoplasmic lysis buffer (10mM HEPES pH 8.0, 10 mM KCl, 1.5 mM MgCl₂, 1 mM DTT, 0.05% Triton X-100, 1X protease inhibitor) with gentle pipetting every 2 minutes during a 10-minute incubation on ice. Lysates were centrifuged at 3000 × g for 5 minutes at 4°C to collect the nuclei pellet, which was resuspended in Percoll solution and centrifuged at 20,000 × g for 15 minutes at 4°C to purify nuclei. The nuclei were then resuspended in nuclear protection buffer (0.32 M Sucrose, 3 mM CaCl_2_, 2 mM MgAC2, 0.1 mM EDTA, 10 mM Tris-HCl, 1 mM DTT, 1X protease inhibitor) and centrifuged at 2500 × g for 3 minutes to remove contaminants. Finally, the nuclei pellet was resuspended in nuclear lysis buffer (20 mM HEPES pH 8.0, 0.42 M NaCl, 1.5 mM MgCl₂, 0.2 mM EDTA, 1 mM DTT, 25% Glycerol, 1X protease inhibitor), incubated for 30 minutes with intermittent pipetting, and centrifuged at 14,000 × g for 10 minutes to collect the supernatant for protein electrophoresis.

*Measurement of serum B2M in mouse*

B2M level in mouse serum was measured via B2M Elisa Kit (Cat. No. ab223590; Abcam, Massachusetts, MA, USA) as per the manufacturer's guidelines. After adding the developing substrate and stopping the reaction, the absorbance at 450 nm was read using Cytation 5 imaging system (BioTek).

*Immunofluorescence and muscle fiber size measurement*

Muscle tissues that had been frozen in an optimal cutting temperature compound were sectioned to a thickness of 10 μm using a cryostat microtome (Leica Microsystems, Wetzlar, Germany) and were stained with laminin-DAPI to measure muscle fiber size. Specifically, the frozen muscle tissues were blocked for 1 h and then incubated with primary antibodies (laminin, 1:1000, Sigma-Aldrich) overnight at 4°C. After washing with PBS, the sectioned tissue slides were incubated with secondary antibodies (Alexa Fluor 647 goat anti-rabbit IgG, 1:1000, Invitrogen) for 1 h at room temperature. Subsequently, they were mounted using a mounting medium with DAPI aqueous fluoroshield. The cross-sectional area (CSA) of muscle fibers was determined by manually drawing contours around individual fibers using ZEN 2 (blue edition) software (Carl Zeiss).

*Skeletal muscle function exploration in mice*

The grip strength test was conducted by securing all four limbs of the mouse on a metal grid and pulling them backward five times. From these trials, the minimal value was excluded, and the remaining four values were averaged. This average was used as the measured value, expressed in Newtons (N). Another method for assessing muscle strength was the Kondziella's inverted screen test. In this test, a weight equivalent to 8-10% of the mouse's body weight was attached to its tail to increase the sensitivity of the test [S2], and the mouse was placed on a wire cage, which was then slowly inverted. The muscle strength of the mice was determined based on the duration they were able to hang onto the wire mesh. The rota-rod performance test involved placing the mouse on a rotating rod and measuring both the distance the mouse traveled before falling and the time it took for the mouse to fall (Jeungdo Bio & Plant Co., Seoul, South Korea). To ensure accurate measurements, the mice underwent acclimatization exercises the day before the actual testing. Each measurement was taken twice with a break in between, rather than continuously, for increased accuracy.

*RNA sequencing analysis*

RNA sequencing, incorporating total RNA isolation, library construction, and FASTQ file creation, was conducted in accordance with protocols by Rokit Genomics, Seoul, Korea. The integrity of the sequencing data was assessed using FastQC v0.11.9. Alignment of FASTQ files to the mouse reference genome (GRCm39, release 109, December 2022) was performed utilizing the Spliced Transcripts Alignment to a Reference (STAR) software v2.7.10a. Gene abundance was quantified employing the RNA-Seq by Expectation-Maximization (RSEM) tool v1.3.1. Normalization of data to adjust for variation due to sequence length and read count was achieved through log2 transformation of Transcripts Per Million (TPM) + 1, enabling robust comparisons across samples. Differential expression analysis was conducted using DESeq2 v1.42.0 with RNA-seq count data. Subsequent analyses, including Gene Set Enrichment Analysis (GSEA), were performed with clusterProfiler v4.10.0, enrichplot v1.22.0, DOSE v3.28.2, and msigdbr v7.5.1. Visualization of RNA sequencing data, encompassing bubble plots for gene set enrichment (Normalized Enrichment Score, NES), and p-values, heatmaps for expression of gene sets, and network plots for gene correlations, were generated through R v4.3.2 and RStudio 2023.03.0 Build 386, utilizing packages including stringr v1.5.1, biomaRt v2.58.0, dplyr v1.1.4, ggpubr v0.6.0, egg v0.4.5, RColorBrewer v1.1-3, readxl v1.3.0, reshape2 v1.4.4, scales v1.3.0, pheatmap v1.0.12, tidyverse v2.0.0, corrr v0.4.4, igraph v2.0.2, ggraph v2.1.0, and tidygraph v1.3.0.

*Label-free 3D live imaging-based mitochondrial dynamics*

AI-powered, label-free 3D live imaging was performed on differentiated myoblasts treated with B2M, with or without ITGB1, using an optical diffraction tomography (ODT) microscope (HT-X1; Tomocube Inc., Daejeon, South Korea). To monitor mitochondrial dynamics, myoblasts were subjected to 3D live imaging following corresponding treatment. Images were captured at 30-second intervals for 1 hour. Quantitative mitochondrial analysis was carried out using holotomography analysis software (TomoAnalysis; Tomocube Inc., Daejeon, South Korea), which automatically identified mitochondria and measured specific parameters following the manufacturer’s protocols.

*Oxygen consumption rates (OCR) measurement*

OCR was measured using a Seahorse XFp Extracellular Flux Analyzer (Seahorse Bioscience, Billerica, MA) according to the manufacturer’s protocol. The OCR measurement was performed using Seahorse XFp Cell Mito Stress Test Kit (103010-100, Seahorse Biosciences), with the following compounds used: oligomycin A (1.5 μM), trifluoromethoxy carbonylcyanide phenylhydrazone (FCCP, 1.5 μM), rotenone (0.5 μM) and antimycin A (0.5 μM). OCR was automatically recorded by the sensor cartridge and calculated using the Seahorse software.

*Evaluation of sarcopenia in older adults*

Experienced nurses conducted interviews and analyzed medical records to gather demographic information and medical histories. Body composition, including muscle mass, were assessed using bioelectrical impedance analysis (InBody S10; InBody, Seoul, South Korea) at frequencies of 1, 5, 50, 250, 500, and 1000 kHz. Appendicular skeletal muscle mass (ASM), representing the combined muscle mass of the upper and lower extremities, was calculated. Additionally, the skeletal muscle mass index (SMI) was computed by dividing ASM by the square of the individual's height (kg/m^2^). Handgrip strength of the dominant arm was measured with a hand dynamometer (Patterson Medical, Warrenville, IL, USA) [S3]. Participants were instructed to sit comfortably, bend their elbows at a 90-degree angle, and grasp the dynamometer as firmly as possible. Two measurements of grip strength were taken, with at least one minute between measurements, and the maximum value was recorded. Gait speed was determined over a 4-meter distance, and the time needed to complete five chair stands was documented [S4]. The short physical performance battery (SPPB) was administered, encompassing assessments of repeated chair stands, standing balance, and gait speed [S5].

The 2019 Consensus Guidelines from the Asian Working Group for Sarcopenia were employed to establish the definition of sarcopenia [S6]. Sarcopenia was diagnosed when individuals presented with low muscle mass combined with weak muscle strength and/or poor physical performance. Low muscle mass was defined as having a SMI of < 7.0 kg/m² for men and < 5.7 kg/m² for women. Weak muscle strength was determined by handgrip strength measurements of less than 28 kg for men and less than 18 kg for women. Poor physical performance was identified by slow gait speed (less than 1.0 m/s), prolonged completion time for the five-time chair stand test (12 seconds or more), or a low SPPB total score (9 points or less).

The sarcopenia phenotype score (SPS) for a continuous measure incorporating multiple features of sarcopenia, which ranged from 0 (best) to 3 (worst), was calculated as the numbers of abnormal parameters among the following three items: low muscle mass, weak muscle strength, and slow gait speed [S7].

*Measurement of serum B2M in older adults*

Blood samples were collected from the antecubital veins of all participants following an overnight fast. Subsequently, the samples underwent centrifugation at 3000 rpm for 5 min at 4°C, resulting in the careful separation of the supernatant to eliminate cellular components. Samples displaying indications of hemolysis or clotting were excluded from the analysis. The obtained serum samples were then stored at -80°C until the quantification of serum B2M concentrations was conducted using the Parameter immunoassay kit (Cat. No. KGE019; R&D Systems, Minneapolis, MN, USA) as per the manufacturer's guidelines. The ELISA kit possessed a lower limit of detection of 0.132 μg/mL, with both intra-assay and inter-assay coefficients of variation measuring below 7.5% and 18.4%, respectively.

**Supplemental References**

S1. Lee JY, Kim DA, Choi E, Lee YS, Park SJ, Kim BJ. Aldosterone Inhibits In Vitro Myogenesis by Increasing Intracellular Oxidative Stress via Mineralocorticoid Receptor. Endocrinol Metab (Seoul). 2021;36:865-74.

S2. Kang JS, Kim D, Rhee J, Seo JY, Park I, Kim JH, et al. Baf155 regulates skeletal muscle metabolism via HIF-1a signaling. PLoS Biol. 2023;21:e3002192.

S3. Roberts HC, Denison HJ, Martin HJ, Patel HP, Syddall H, Cooper C, et al. A review of the measurement of grip strength in clinical and epidemiological studies: towards a standardised approach. Age Ageing. 2011;40:423-9.

S4. Peel NM, Kuys SS, Klein K. Gait speed as a measure in geriatric assessment in clinical settings: a systematic review. J Gerontol A Biol Sci Med Sci. 2013;68:39-46.

S5. Jung HW, Roh H, Cho Y, Jeong J, Shin YS, Lim JY, et al. Validation of a Multi-Sensor-Based Kiosk for Short Physical Performance Battery. J Am Geriatr Soc. 2019;67:2605-9.

S6. Chen LK, Woo J, Assantachai P, Auyeung TW, Chou MY, Iijima K, et al. Asian Working Group for Sarcopenia: 2019 Consensus Update on Sarcopenia Diagnosis and Treatment. J Am Med Dir Assoc. 2020;21:300-7.e2.

S7. Ahn SH, Jung HW, Lee E, Baek JY, Jang IY, Park SJ, et al. Decreased Serum Level of Sclerostin in Older Adults with Sarcopenia. Endocrinol Metab (Seoul). 2022;37:487-96.
