# Supplementary material for: Detrimental Effects of β2‐Microglobulin on Muscle Metabolism: Evidence From In Vitro, Animal and Human Research
Source: J Cachexia Sarcopenia Muscle. 2025 Mar 3;16(2):e13745. doi: 10.1002/jcsm.13745 (PMC11873540; doi:10.1002/jcsm.13745)
Supplement: Supplementary file 2 — Figure S1 B2M levels increase with ageing. (a) B2M levels exhibit an age‐dependent increase in skeletal muscle from both human and murine models. (b) Serum B2M levels increase in aged mice. B2M, β2‐Microglobulin. *p < 0.05 vs. young mice. Figure S2. Western blot analyses of β‐tubulin and Histone H3 in nucleus and cytosol. B2M, β2‐microglobulin; TS2/16, ITGB1‐activating antibody. Figure S3. Serum B2M levels exhibited a 5.9‐fold increase following systemic B2M in mice. Three‐month‐old male mice were intraperitoneally injected with PBS (100 μL) or recombinant B2M (250 μg/100 μL) for 4 weeks (n = 6 per group). B2M, β2‐microglobulin; PBS, phosphate‐buffered saline. *p < 0.05 vs. untreated control or before treatment. Figure S4. B2M treatment does not alter the proportions of Type I, Type IIa and Type IIb fibres in the tibialis anterior and soleus muscles. Three‐month‐old male mice were intraperitoneally injected with PBS (100 μL) or recombinant B2M (250 μg/100 μL) for 4 weeks (n = 6 per group). Representative images of immunofluorescent staining for laminin (green) and MyHC I, MyHC IIa and MyHC IIb (red) are shown. The relative frequency of each fibre type in the tibialis anterior and soleus muscles was evaluated. Scale bars: 100 μm. B2M, β2‐microglobulin; MyHC, myosin heavy chain; PBS, phosphate‐buffered saline. Figure S5. Bubble plots summarizing B2M‐induced gene sets alteration identified by gene set enrichment analysis. B2M, β2‐microglobulin. Figure S6. B2M impairs mitochondrial metabolism via ROS production. (a,b) Quantitative reverse‐transcription polymerase chain reaction of Ppargc1a and Tfam in vivo (a) and in vitro (b) (n = 3). (c,d) Mitochondrial function analysis via oxygen consumption rate in differentiated myotube, treated with or without B2M and/or NAC. Ant, antimycin; FCCP, carbonyl cyanide‐p‐trifluoromethoxyphenylhydrazone; Oligo, oligomycin; Rot, rotenone. (e–i) Quantitative characteristics of mitochondria in differentiated myotube, treated with or witho [file JCSM-16-e13745-s002.docx]

**
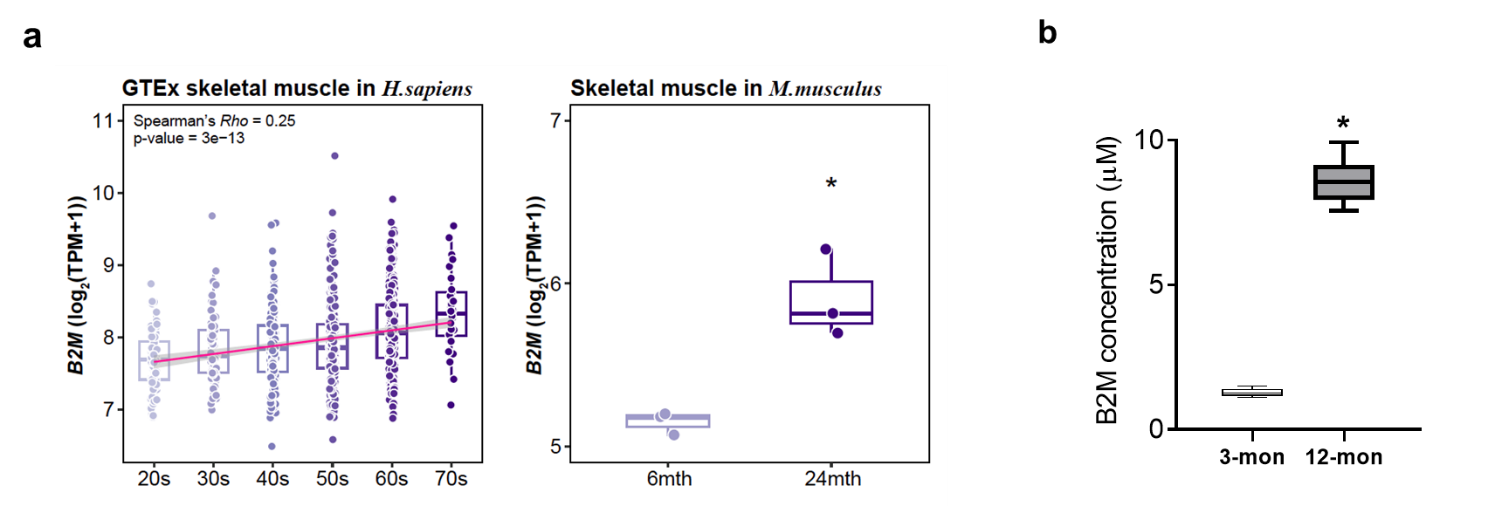
**

**Figure S1.** B2M levels increase with aging. (a) B2M levels exhibit an age-dependent increase in skeletal muscle from both human and murine models. (b) Serum B2M levels increase in aged mice. B2M, β2-Microglobulin. **P* < 0.05 vs. young mice.

**
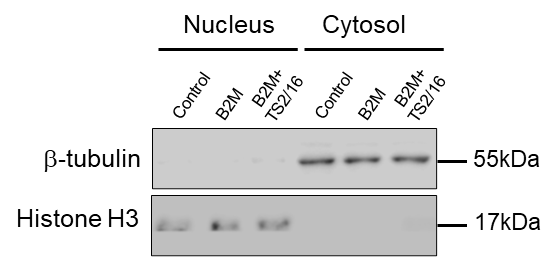
**

**Figure S2.** Westen blot analyses of β-tubulin and Histone H3 in nucleus and cytosol. B2M, β2-Microglobulin; TS2/16, ITGB1-activating antibody

**Figure S3.** Serum B2M levels exhibited a 5.9-fold increase following systemic B2M in mice. Three-month-old male mice were intraperitoneally injected with PBS (100 μL) or recombinant B2M (250 μg/ 100 μL) for 4 weeks (*n* = 6 per group). B2M, β2-Microglobulin; PBS, phosphate-buffered saline. B2M, β2-Microglobulin. **P* < 0.05 vs. untreated control or before treatment.


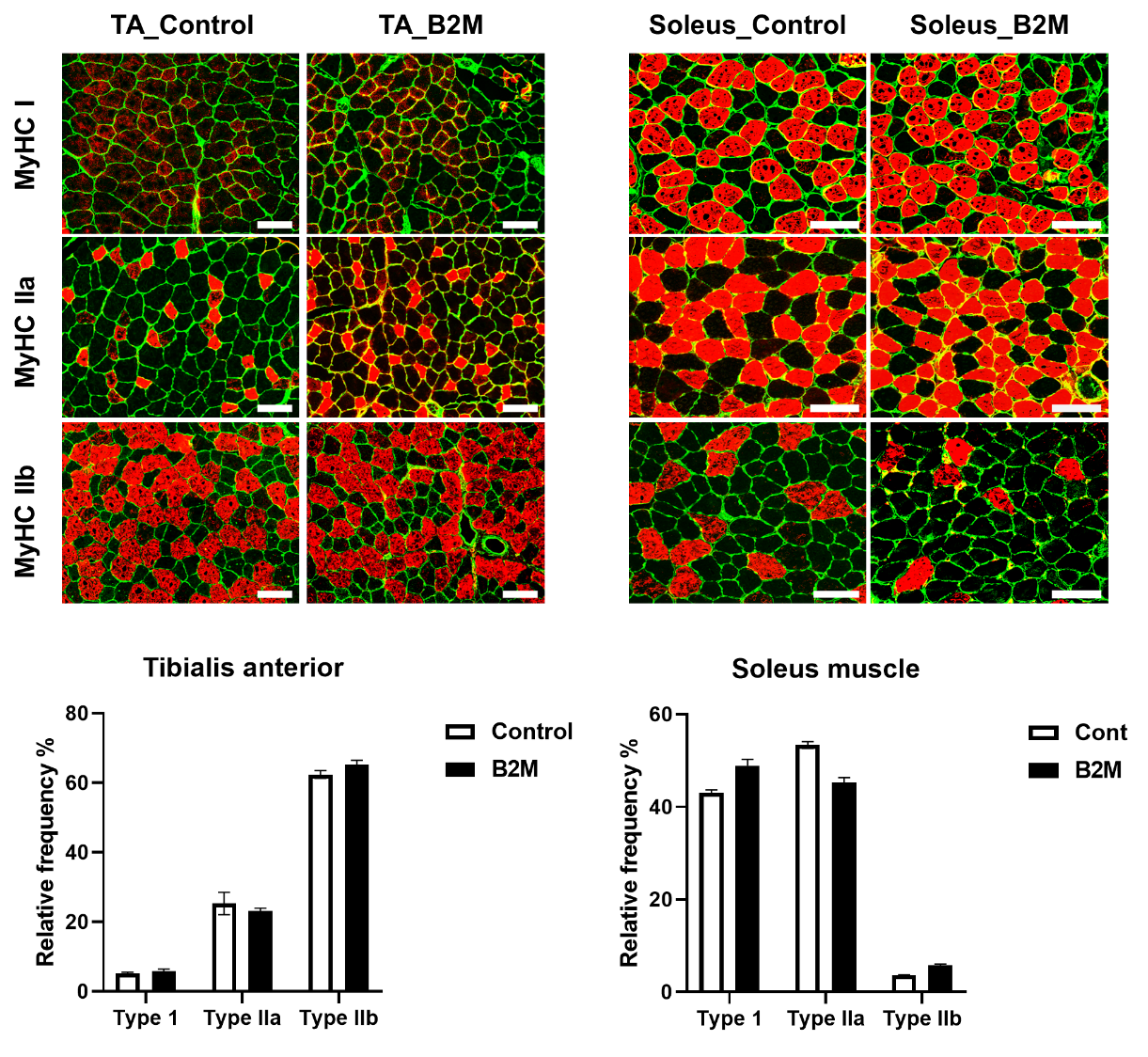


**Figure S4.** B2M treatment does not alter the proportions of type I, type IIa, and type IIb fibers in the tibialis anterior and soleus muscles. Three-month-old male mice were intraperitoneally injected with PBS (100 μL) or recombinant B2M (250 μg/100 μL) for 4 weeks (*n* = 6 per group). Representative images of immunofluorescent staining for laminin (green) and MyHC I, MyHC IIa, and MyHC IIb (red) are shown. The relative frequency of each fiber type in the tibialis anterior and soleus muscles was evaluated. Scale bars: 100 μm. B2M, β2-Microglobulin; PBS, phosphate-buffered saline; MyHC, myosin heavy chain.


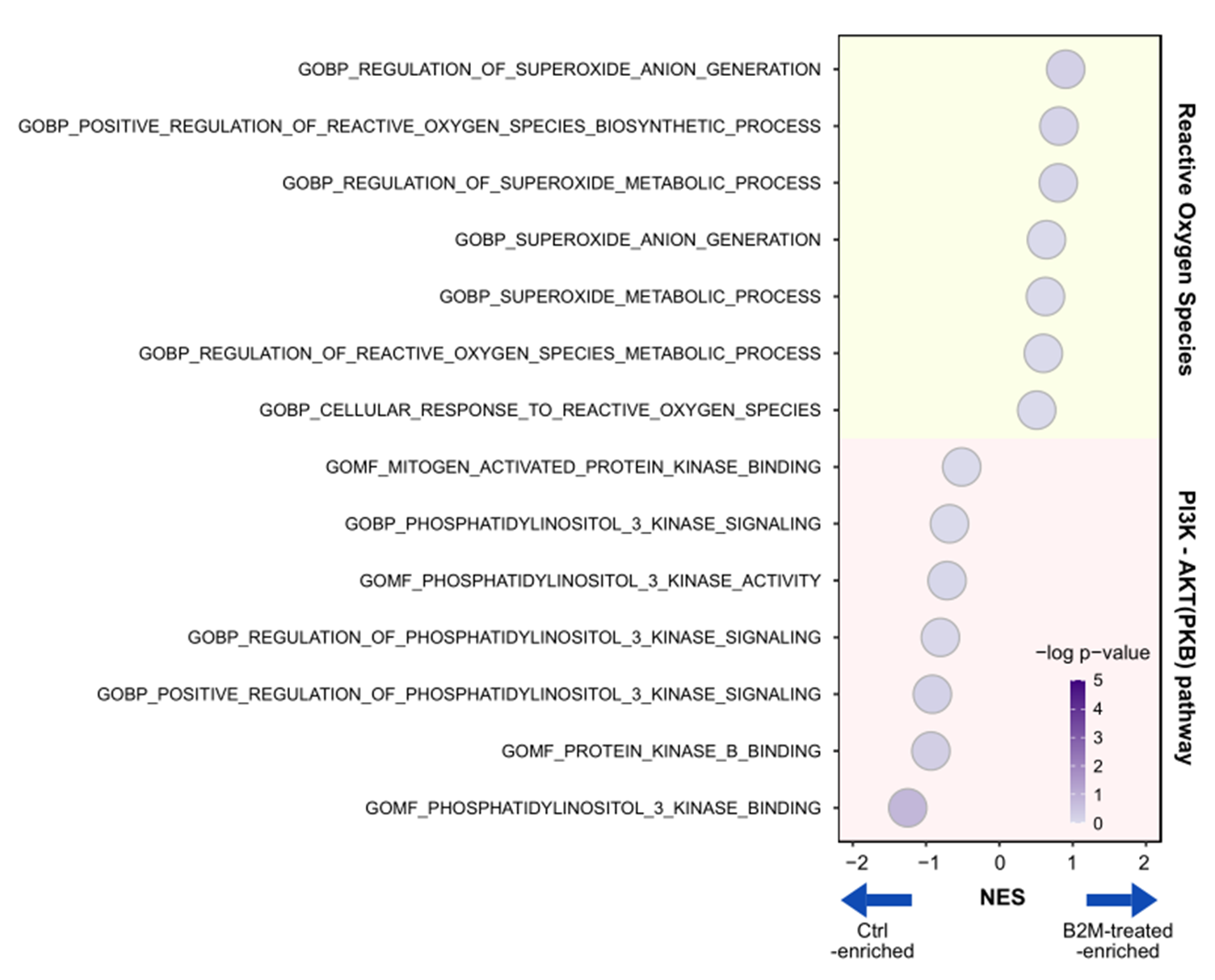


**Figure S5.** Bubble plots summarizing B2M-induced gene sets alteration identified by gene set enrichment analysis. B2M, β2-Microglobulin.


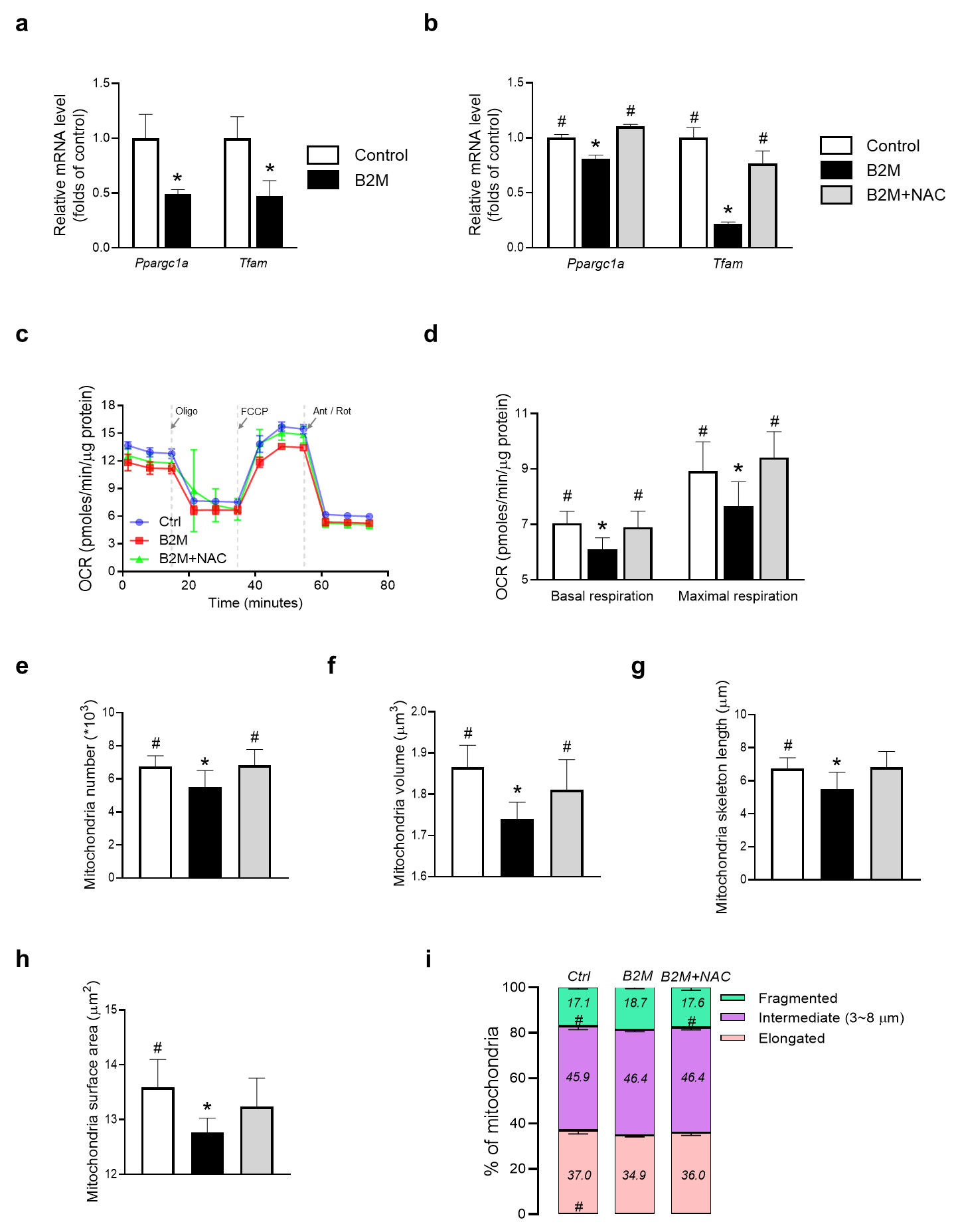


**Figure S6.** B2M impairs mitochondrial metabolism via ROS production. (a and b) Quantitative reverse-transcription polymerase chain reaction of Ppargc1a and Tfam in vivo (a) and in vitro (b) (*n* = 3). (c and d) Mitochondrial function analysis via oxygen consumption rate in differentiated myotube, treated with or without B2M and/or NAC. Oligo: oligomycin, FCCP: Carbonyl Cyanide-p-trifluoromethoxyphenylhydrazone, Ant: antimycin, Rot: rotenone. (e-i) Quantitative characteristics of mitochondria in differentiated myotube, treated with or without 10 μM B2M and/or 1 mM NAC. Parameters analyzed include mitochondrial number (e), volume (f), skeleton length (g), and surface area (h). Mitochondrial dynamics were assessed by calculating the percentage of mitochondria (i) exhibiting morphological features based on skeleton length: elongation (≥8 μm), intermediate (3~8 μm), fragmentation (<3 μm) (*n* = 8). B2M, β2-Microglobulin; OCR: oxygen consumption rate. **P* < 0.05 vs. untreated control; #*P* < 0.05 vs. 10 μM B2M.

**
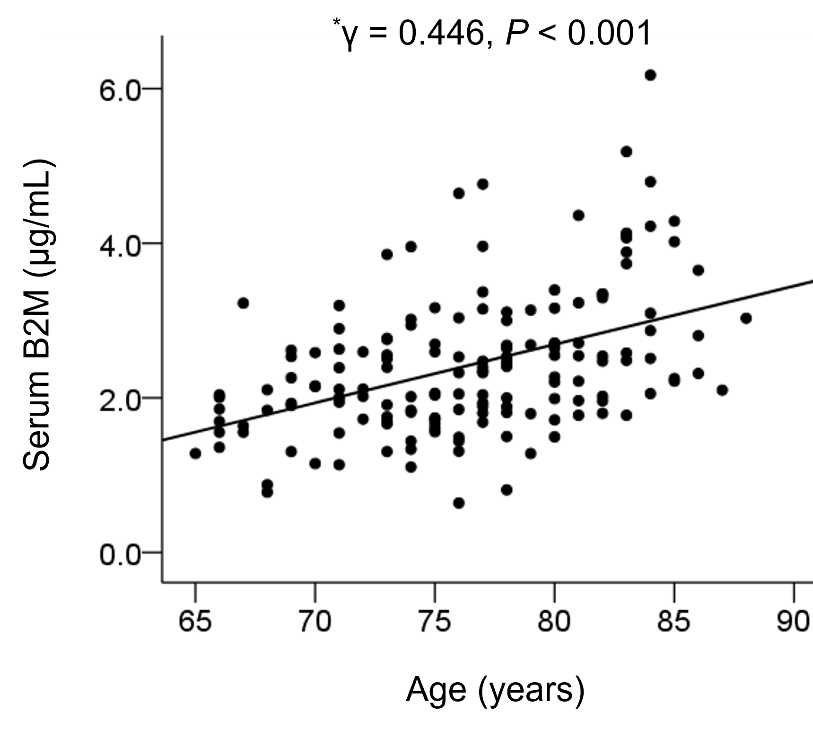
**

**Figure S7.** Pearson correlation coefficient with scatter plots for the association of age with serum B2M level. * indicates a statistically significant value. B2M, β2-microglobulin.

**Table S1.** Basic clinical characteristics of the study participants

|  | No sarcopenia  (n = 118) | Sarcopenia  (n = 40) | *p* |
| --- | --- | --- | --- |
| Age, y | **75.3 ± 5.2** | **79.7 ± 4.6** | **<0.001** |
| Female | 97 (82.2) | 29 (72.5) | 0.254 |
| Body weight, kg | **59.4 ± 9.6** | **53.6 ± 5.5** | **<0.001** |
| Height, cm | 155.0 ± 6.5 | 153.2 ± 6.9 | 0.157 |
| Body mass index, kg/m^2^ | **25.4 ± 5.5** | **22.9 ± 2.5** | **0.007** |
| Diabetes mellitus | 46 (39.0) | 13 (32.5) | 0.571 |
| Polypharmacy | 59 (50.0) | 24 (60.0) | 0.360 |
| Fall in previous year | 21 (17.8) | 8 (20.0) | 0.814 |
| Appendicular skeletal muscle mass, kg | **15.2 ± 2.9** | **13.2 ± 2.3** | **<0.001** |
| Skeletal muscle mass index, kg/m^2^ | **6.30 ± 0.79** | **5.56 ± 0.52** | **<0.001** |
| Grip strength, kg | **25.9 ± 6.3** | **19.9 ± 5.1** | **<0.001** |
| Usual gait speed, m/s | **1.04 ± 0.24** | **0.77 ± 0.26** | **<0.001** |
| Chair stand test time, s | **10.4 ± 5.5** | **17.3 ± 13.5** | **0.003** |
| SPPB total score (ranges, 0-12) | **10.9 ± 1.6** | **8.7 ± 3.1** | **<0.001** |
| SPS score (range, 0-3) | **0.73 ± 0.57** | **2.40 ± 0.50** | **<0.001** |
| Serum B2M, μg/mL | **2.26 ± 0.87** | **2.62 ± 0.86** | **0.023** |

Data are presented as mean ± standard deviation or n (%). Differences between the two groups were assessed using Student’s *t-*tests for continuous variables and χ^2^ test for categorical variables. SPPB, short physical performance battery; SPS, sarcopenia phenotype score. B2M, β2-microglobulin.
